# Histone H2B-associated proteins: The Arabidopsis nucleolin 1 binds H2B and facilitates nucleosome disassembly *via* RNA-dependent mechanism

**DOI:** 10.1016/j.jbc.2026.113137

**Published:** 2026-05-11

**Authors:** Naveen Kumar Yarra, Jeevan R. Singiri, Zachor Agmon-Adler, Rohith Grandhi, Asha Sastya, Nurit Novoplansky, Gideon Grafi

**Affiliations:** French Associates Institute for Agriculture and Biotechnology of Drylands, Jacob Blaustein Institutes for Desert Research, Ben-Gurion University of the Negev, Be'er Sheva, Israel

**Keywords:** Arabidopsis thaliana, ATP-dependent nucleosome remodeling factors, gene transcription, H2B-interacting proteins, histone H2B variants, nucleolin 1 (AtNuc-L1), nucleosomes, nucleosome disassembly, RNA recognition motif (RRM)

## Abstract

To gain insight into the function of histone H2B variants in Arabidopsis, we studied the histone H2B.9 variant, attempting to uncover its interacting proteins. Accordingly, nuclear extract derived from H2B.9-GFP–expressing plants was subjected to GFP-Trap followed by proteome analysis. This analysis revealed 106 H2B.9-associated proteins, among them splicing factors, chromatin remodeling factors, and nucleolar proteins, including the histone chaperone nucleolin 1 (AtNuc-L1). Like animal nucleolins, AtNuc-L1 is known for its function in rRNA transcription and ribosome biogenesis, as well as its role in chromatin organization and nucleosome sliding; yet, the molecular mechanism underlying its function remains unclear. We showed that it specifically binds histone H2B, but not other core histones, in the context of nucleosomes and facilitates disassembly of naturally occurring nucleosomes derived from tobacco leaves *via* chromatin fractionation. This activity requires additional nuclear factors and is dependent on binding RNA molecules. Thus, our findings reveal the magnitude of nuclear processes potentially mediated by histone H2B and provide evidence for the role of the H2B-interacting AtNuc-L1 in RNA-dependent nucleosome disassembly.

The higher-order packaging of chromatin influences the accessibility of the genetic material for nuclear processes such as replication, DNA repair, and gene expression, which is essential for maintaining the cellular identity (1, 2). The chromatin is highly dynamic and can be extensively remodeled by posttranslational modifications of histones and the incorporation of histone variants, facilitated by histone chaperones and ATP-dependent nucleosome remodeling factors (3). Histone variants are nonallelic protein isoforms encoded by paralogous genes that share a structural domain called the histone fold, yet their diverse roles are not fully understood. Beyond their primary role in DNA packaging, core histone proteins act as an essential hub controlling various nuclear processes, including gene transcription, splicing, DNA replication, and DNA repair (4). Among the core histone proteins, the histone H2B variants found in plants exhibit the least conservation, primarily due to variations in their N-terminal regions (5, 6, 7). The model plant Arabidopsis has 11 genes encoding for histone H2B proteins that are divided into three phylogenetic clusters termed class I, II, and III (6). Generally, class I variants, specifically H2B.9 and H2B.4 (nomenclature by (8)), are highly expressed in *Arabidopsis* leaves, while class II and class III are particularly expressed in reproductive organs (6).

In order to understand the extent of nuclear activities governed by the class I variant H2B.9, we aimed to identify its interacting proteins. GFP-TRAP of nuclear proteins from H2B.9-GFP–expressing plants (9) coupled with proteome analysis revealed multiple nuclear proteins, among them splicing factors, chromatin remodeling factors, and many nucleolar proteins including the histone chaperone nucleolin 1 (AtNuc-L1), which represents the focus of this study. Nucleolins are abundant proteins localized mainly in the nucleolus and can be found in a variety of organisms, including yeast, plants, and mammals. Nucleolins from various eukaryotes share structural similarity, and all have three characteristic domains, namely, an acidic N-terminal region, a central region that contains two or four RNA recognition motifs (RRMs), and the C-terminal GAR domain, rich in glycine and arginine (10). Nucleolins have been particularly studied with respect to nucleolus structure, rRNA gene transcription, and ribosome biogenesis (10), but their function is much broader, affecting cellular processes such as DNA and RNA metabolism, chromatin remodeling, and cell cycle regulation (11, 12); they also emerged as key players in multiple pathologies, particularly cancer, where they contribute to tumor growth, metastasis, and drug resistance (13, 14). Early work in animal cells showed that nucleolin is loosely associated with ribonucleoprotein complexes and appears to be present in the nucleosome fraction (15). It has been shown to bind the H2A/H2B dimer, facilitating transcription through nucleosomes (16, 17), as well as binding to histone H1 to induce chromatin decondensation (18). Nucleolins are also involved in the compartmentalization, stability, and dynamics of H2B within the nucleolus (19). AtNuc-L1 is an Arabidopsis protein localized mainly to the nucleolus, the site of rRNA gene transcription, and plays a vital role in ribosome biogenesis, nucleolus structure, chromosome organization, and nucleosome sliding and consequently in plant growth, development, and patterning (20, 21, 22, 23, 24). The present study revealed the H2B.9-associated proteins and focuses on the H2B.9-interacting nucleolin 1 (AtNuc-L1) and the molecular mechanism underlying its function in promoting the disassembly of nucleosomes.

## Results

### Exploring the H2B.9-associated proteins: GFP-TRAP

We used transgenic Arabidopsis plants expressing H2B.9-GFP (9) for identifying H2B.9-interacting proteins using the GFP-TRAP method. To this end, total nuclear proteins extracted from H2B.9-GFP–expressing plants were subjected to GFP-TRAP followed by proteomic analysis. As a control, we used nuclear extracts derived from Arabidopsis plants expressing the nuclear roGFP2-ORP1 protein. After filtering out potential contaminants and retaining only proteins identified by at least two peptides and present in at least two replicates of at least one group, 436 proteins were recovered (Table S1). Further filtering for nuclear proteins present in at least two replicates of the H2B.9-GFP group and not present or present in only one replicate of the control roGFP2-ORP1 group revealed 106 nuclear proteins that associate with H2B.9 (Tables 1 and S2). Thus, numerous proteins appear to be associated with histone H2B.9 that can be classified into several groups, including splicing factors, among them the apoptosis and splicing associated proteins, SAP18, SR45, and PININ. In addition, H2B.9 associates with ATP-dependent chromatin remodeling factors (*e.g.*, SWI3A, SWI3B, CHR4), transcription factors (FGT1, HDA15, NRPB3), nuclear lamina proteins (CRWN1, CRWN3), DNA repair (PDS5A, PDS5C), and multiple nucleolar proteins (PRH75, FIB1, AtNUC-L1). AtNuc-L1, like yeast and mammalian nucleolins, acts as a histone chaperone implicated in ribosome biogenesis and genome organization (23, 25), but its molecular function is unclear.

**Table 1:** Partial list of H2B.9-associated proteins, which are present in H2B.9-GFP and “absent” in the reference roGFP2-ORP1-1 precipitates (The complete list is given in Table S2)

Gene IDs	Protein names	Gene names
Splicing factors		
AT1G16610	Arginine/serine-rich 45 (ASAP complex)	SR45
AT3G19760	Eukaryotic initiation factor 4A-III homolog	EIF4A3
AT1G15200	PININ (ASAP complex)	At1g15200
AT3G49601	Pre-mRNA-splicing factor	At3g49601
AT2G45640	SAP18 (ASAP complex)	SAP18
Chromatin remodeling factors		
AT3G06010	Probable ATP-dependent DNA helicase CHR12	CHR12
AT3G57300	Chromatin-remodeling ATPase INO80	INO80
AT2G19480	Nucleosome assembly protein 12	NAP1
AT5G44800	Protein CHROMATIN REMODELING 4	CHR4
AT2G47620	SWI/SNF complex subunit SWI3A	SWI3A
AT2G33610	SWI/SNF complex subunit SWI3B	SWI3B
Transcription factors		
AT1G33240	GT-2-like 1;Trihelix transcription factor GTL1	GTL1
AT4G00270	GLABROUS1 enhancer-binding protein	GEBP
AT5G25220	Homeobox protein knotted-1-like 3	KNAT3;KNAT4
AT1G76110	High mobility group B protein 9	HMGB9
AT3G18520	Histone deacetylase 15	HDA15
AT1G79350	Protein FORGETTER 1	FGT1
AT2G15430	DNA-directed RNA polymerases II, IV, and V subunit 3	NRPB3
Nuclear lamina		
AT1G67230	Protein CROWDED NUCLEI 1	CRWN1
AT1G68790	Protein CROWDED NUCLEI 3	CRWN3
DNA repair		
AT5G47690	Sister chromatid cohesion protein PDS5 homolog A	PDS5A
AT4G31880	Sister chromatid cohesion protein PDS5 homolog C	PDS5C
Nucleolar proteins/ribosome biogenesis		
AT1G50920	Nucleolar GTP-binding protein 1	At1g50920
AT1G56110	Nucleolar protein 56	NOP56
AT1G48920	**Nucleolin 1**	**NUCL1**

### AtNuc-L1 binds H2B in the context of nucleosomes

**Figure 1:**
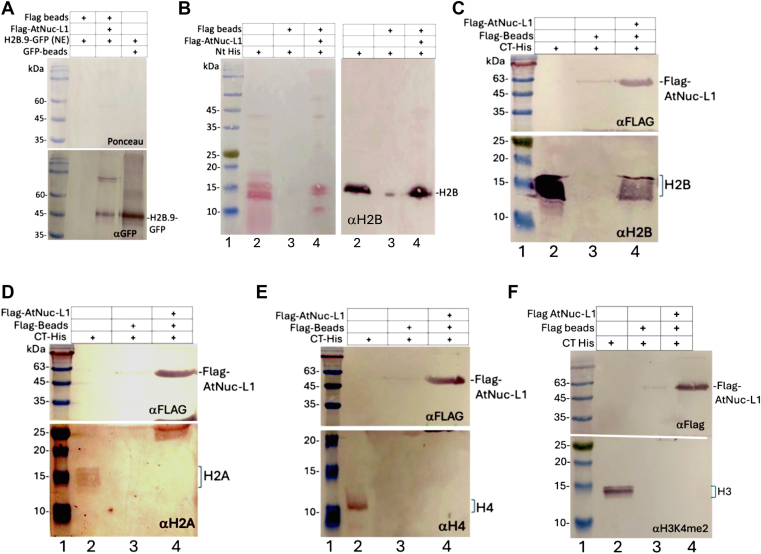
**The AtNuc-L1 protein specifically interacts with H2B.***A*, Flag-AtNuc-L1 immobilized on Flag beads or GFP-TRAP beads were incubated with nuclear extract derived from H2B.9-GFP–expressing plants. Precipitated proteins were separated on 12% SDS/PAGE and immunoblotted with αGFP. *Upper* panel is ponceau staining and lower panel is immunoblot. *B*, AtNuc-L1 interacts with histone H2B from tobacco histone preparation (Nt His). Lane 2 is the input histones. *Left* panel is Ponceau staining and *right* panel is western with αH2B. *C*, AtNuc-L1 interacts specifically with H2B from calf thymus preparation (CT-His), but not with H2A (*D*), H4 (*E*), or histone H3 (*F*). Lane 2 in each panel is the input histones. Note, each membrane in (*C*) to (*F*) was cut above the 20 to 25 kDa to probe the *upper* part and the *lower* part with anti-Flag and the indicated anti-histone, respectively. Also note, the *lower* part in (*D*) was color adjusted to increase visibility of the input H2A signal.

**Figure 2:**
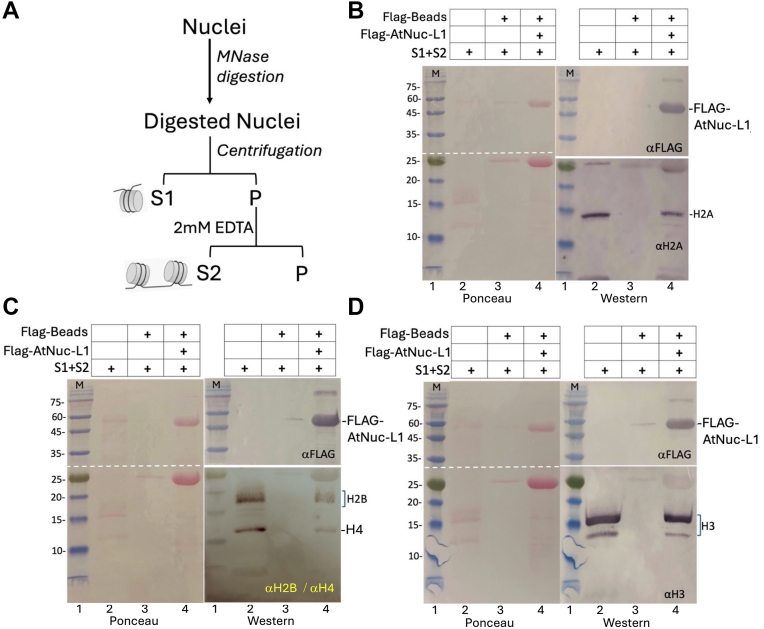
**AtNuc-L1 binds histone H2B in the context of nucleosomes.***A*, schematic depiction of chromatin fractionation for obtaining nucleosomes (fraction S1+S2). Nucleosomes were incubated with Flag beads or with Flag-AtNuc-L1 immobilized on Flag-beads and precipitates were subjected to immunoblotting using anti-H2A (*B*), anti-H2B and anti-H4 (*C*), and anti-H3 (*D*). Ponceau staining of the membrane is shown on the *left* in each panel. Each membrane was cut above the 25 kDa to probe the *upper* part and the *lower* part with anti-Flag and the indicated anti-histone protein, respectively. Note, (*C*) was first probed with αH2B followed by reprobing with αH4. In each (*B–D*), lane 2 refers to the input S1+S2 fractions.

Focusing on AtNuc-L1, we sought to verify its interaction with histone H2B. To this end, the Flag-AtNuc-L1 DNA sequence was subcloned into pET28a, and the Flag-AtNuc-L1 protein (devoid of the HIS tag) was expressed in bacteria. Purification on Flag beads revealed two major proteins, one at the expected size of Flag-AtNuc-L1 of about 60 kDa and another protein of about 25 kDa (Fig. 2, *B*–*D*). Flag-AtNuc-L1 immobilized on Flag beads pulled down H2B.9-GFP from nuclear extract derived from H2B.9-GFP–expressing plants (Fig. 1*A*) or H2B from tobacco histone preparation (Nt His, Fig. 1*B*), but not H2A or H3 (Fig. S1). Similar results were obtained with histones from calf thymus (CT His) showing that Flag-AtNuc-L1 interacts specifically with H2B (Fig. 1*C*), but not with other core histone proteins, namely H2A (Fig. 1*D*), H4 (Fig. 1*E*), or H3 (Fig. 1*F*). Finally, a reciprocal experiment showed that anti H2B immobilized onto protein A-agarose in the presence of CT His–precipitated Flag-AtNuc-L1 from bacterial extract (Fig. S1*D*).

We examined the capacity of AtNuc-L1 to bind histone H2B in the context of nucleosomes. To this end, we used native nucleosomes extracted from tobacco leaves by chromatin fractionation methodology (Fig. 2*A*). Accordingly, nucleosomes-containing S1 and S2 fractions were incubated with Flag-AtNuc-L1 immobilized onto anti-Flag-beads and precipitated nucleosomes were run on 15% SDS/PAGE, transferred onto membrane (Fig. 2, *B*–*D*, left panels), and the upper part (above 25 kDa) and the lower part (below 25 kDa) were immunoblotted with anti-Flag and with the indicated anti-histones, respectively. Thus, the results showed that AtNuc-L1 can pull down intact nucleosomes since the examined core histone proteins, H2A (Fig. 2*B*), H2B and H4 (Fig. 2*C*), and H3 (Fig. 2*D*) were recovered in the Flag-AtNuc-L1 immunoprecipitates.

### AtNuc-L1 facilitates nucleosome disassembly

**Figure 3:**
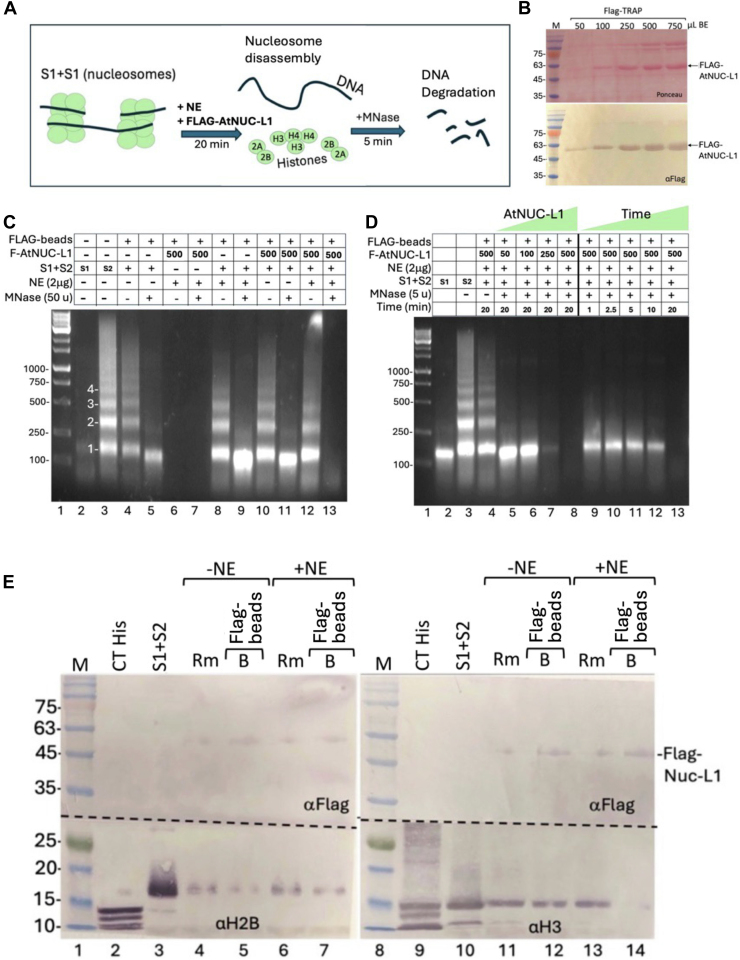
**AtNuc-L1 function in nucleosome disassembly.***A*, illustration of the method used for the analysis of nucleosome disassembly. S1+S2 nucleosome-containing fractions were incubated with nuclear extract (NE) and Flag-AtNuc-L1 (F-AtNuc-L1, given in μl bacterial extract) for 20 min at room temperature followed by incubation with MNase for 5 min. Nucleosome disassembly separates DNA from core histones exposing it to degradation by MNase. *B*, purification of Flag-AtNuc-L1 by Flag-TRAP. Various amounts of bacterial extract containing Flag-AtNuc-L1 were incubated with Flag beads; bound proteins were run on 12% SDS/PAGE and immunoblotted using anti-Flag (αFlag). *C*, AtNuc-L1 is required for nucleosome disassembly activity. Each reaction mixture (lanes 2–13) contains the indicated components. Flag beads, Flag-AtNuc-L1 containing bacterial extract (500 μl), nuclear extract (NE, 2 μg), S1+S2 fraction (equivalent to 1 g leaf tissue), and Micrococcal nuclease (MNase 50 u). *D*, kinetics of nucleosome disassembly and DNA exposure for MNase degradation as determined by increasing levels of Flag-AtNuc-L1 (lanes 5–8) or increasing incubation time (lanes 9–13). *E*, confirmation of nucleosome disassembly by AtNuc-L1. Reactions were conducted in the presence of S1+S2 and Flag-AtNuc-L1 either with (+NE) or without (−NE) nuclear extract followed by immunoprecipitation with Flag-beads. Lanes 2 and 9 are reference histone preparation from calf thymus (CT His) and lanes 3 and 10 are the input S1+S2 fractions. Lanes 4, 6, 11, and 13 represent the input reaction mixtures (Rm). Bound (B) proteins (lanes 5, 7, 12, and 14) were separated on 12% SDS/PAGE, transferred onto membrane and immunoblotted. Note, each membrane was cut above the 25 kDa (broken line) to probe the *upper* part and the *lower* part with anti-Flag and the indicated anti-histone protein, respectively.

The nucleolin proteins in mammals functions as histone chaperones assisting the activity of SWI/SNF remodeling factor in destabilization of the histone octamer; it can remove H2A–H2B dimers from assembled nucleosomes and facilitates transcription (16). The finding that H2B.9-GFP can bind chromatin remodeling factors such as SWI3A and CHR4 together with AtNuc-L1 prompted us to address its capacity to disassemble nucleosomes. Thus, we assumed that upon nucleosome disassembly, core histones are separated from the DNA, making it susceptible to digestion by micrococcal nuclease (Fig. 3*A*). We wanted to address the capacity of AtNuc-L1 produced as a Flag-AtNuc-L1 (Fig. 3*B*) to disassemble genuine nucleosomes rather than an artificial nucleosomal array. Thus, nucleosome fractions were obtained from tobacco leaves by using chromatin fractionation (Fig. 2*A*). Nuclei prepared from tobacco leaves were treated with micrococcal nuclease to yield the S1 fraction containing mainly mononucleosomes and the S2 fraction containing oligonucleosomes (Fig. 3*C*, lanes 2 and 3), which are demonstrated by the DNA ladder protected by one or more nucleosomes. A mix of nuclear extracts (NE) from Arabidopsis and tobacco leaves was added to the reaction mixture as a source for remodeling factors required for nucleosome disassembly. Accordingly, when the reaction mixture is lacking, Flag-At-NUC-L1 (Fig. 3*C*, lanes 2–5, 8, 9) or NE (lanes 2–5, 10, 11) disassembly did not occur. However, in the presence of all components, namely, Flag-AtNuc-L1, NE and S1+S2 (lanes 12, 13) nucleosome disassembly occurs. Thus, in the absence of MNase, nucleosomal DNA was fully recovered (lane 12) but completely lost when MNase was added (lane 13), indicating that AtNuc-L1 in the presence of NE allows for nucleosome disassembly to occur. To further verify this function of AtNuc-L1, we address the kinetic of DNA degradation by MNase under various levels of Flag-AtNuc-L1 or under various incubation time. Thus, nucleosome disassembly has gradually occurred as evidenced by degradation of the DNA as the level of Flag-AtNuc-L1 (Fig. 3*D*, lanes 5–8) and incubation time (Fig. 3*D*, lanes 9–13) were increased (Fig. 3*D*). These results confirmed the function of AtNuc-L1 in nucleosome disassembly.

To confirm that the disassembly of nucleosomes was facilitated by Flag-AtNuc-L1 rather than by copurifying proteins, we conducted the assay using a reference Flag-Krüppel-like factor 4 (KLF4) transcription factor protein (26) or copurifying proteins obtained from bacteria harboring the parental pET28a (Fig. S2*A*). The disassembly of nucleosomes was clearly observed with Flag-AtNuc-L1 (Fig. S2*B*, lanes 7 and 8); however, no activity was detected with the copurifying proteins from pET28a (Fig. S2*B* lanes 3 and 4) or with the Flag-KLF4 protein (Fig. S2*B* lanes 5 and 6).

We confirmed disassembly of the nucleosome rather than sliding by immunoprecipitation. Thus, following the completion of reaction, the reaction mixture (Rm) was subjected to immunoprecipitation using Flag-TRAP. If the octamer is preserved (sliding), we should recover both H2B and H3, but if the octamer disintegrates (disassembly), we should recover only H2B but not H3. Results showed (Fig. 3*E*, see also Fig. S3) the recovery of H2B and H3 from S1+S2 fractions (Fig. 3*E*, lanes 3 and 10) and from NE-deficient reaction mixture containing FLAG-AtNuc-L1 (lanes 4, 5 and 11, 12). However, in the reaction mixture containing NE and Flag-AtNuc-L1, H2B (Fig. 3*E*, lane 7), but not H3 (Fig. 3*E*, lane 14), was recovered following immunoprecipitation with anti-Flag, suggesting that the nucleosomal octamer was not preserved and underwent disassembly.

### Nucleosome disassembly requires ATP

**Figure 4:**
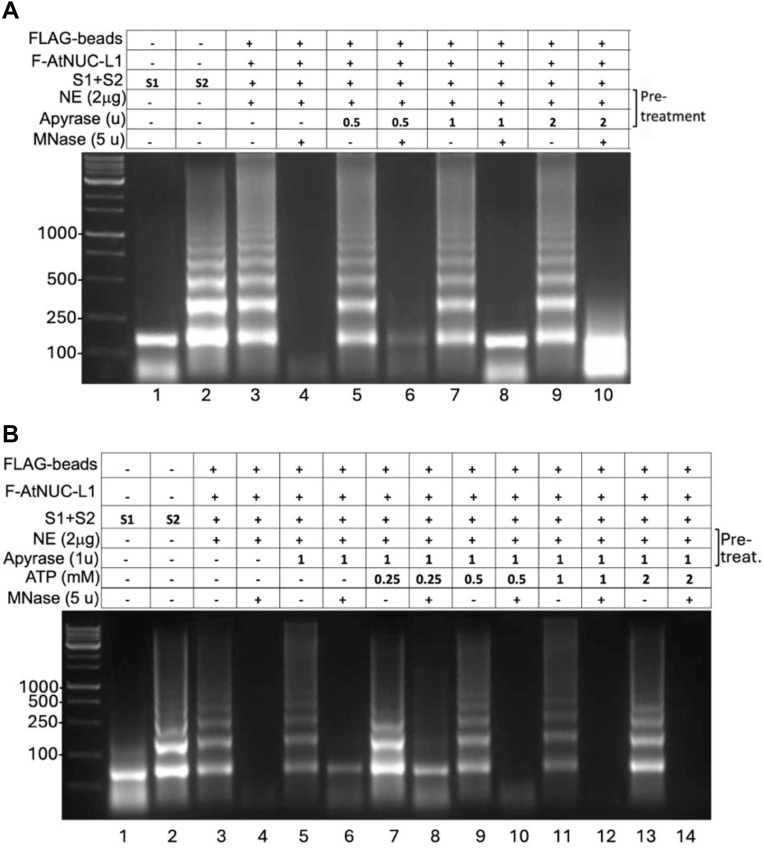
**Nucleosome disassembly requires ATP.***A*, ATP removal from nuclear extract (NE) by apyrase abolished nucleosome disassembly. NE pretreated with apyrase (0.5, 1, and 2 units, lanes 5–10) was added to the reaction mixture followed by the extraction of nucleosomal DNA. *B*, adding ATP to apyrase-pretreated NE restored nucleosome disassembly activity. Reaction mixture containing apyrase (1 unit) pretreated-NE was added various concentrations of ATP (0.25, 0.5, 1, 2 mM; lanes 7–14) and the extent of nucleosome disassembly was monitored following nucleosomal DNA extraction. Note, adding 0.5 mM ATP was sufficient for full recovery of nucleosome disassembly.

Given that the disassembly of nucleosomes requires the function of the ATP-dependent SWI/SNF nucleosome-remodeling factor (16), and considering that H2B.9 is associated with ATP-dependent chromatin-remodeling factors (*e.g.*, SWI3A, SWI3B), we aimed to explore the need for ATP in the process of AtNuc-L1–mediated nucleosome disassembly. Accordingly, NE, which serves as a source for nucleosome-remodeling factors was treated with the enzyme apyrase that hydrolyses ATP and ADP to produce AMP and inorganic phosphates. Apyrase-treated NE was then added to the reaction mixture containing S1+S2, Flag beads, and Flag-AtNuc-L1 followed by incubation with MNase. These experiments showed that in contrast with untreated NE, pretreatment of NE with 0.5 unit apyrase slightly reduced nucleosome disassembly as shown by the recovery of low amount of DNA (Fig. 4*A*, lane 6). However, under high concentrations of apyrase (1 and 2 units), nucleosome disassembly was completely abolished and DNA was fully recovered (Fig. 4*A*, lanes 8 and 10). To verify the requirement for ATP, we performed reciprocal experiments by adding ATP to the reaction mixture containing apyrase-treated NE (Fig. 4*B*). The results showed the addition of 0.5 mM ATP was sufficient for full recovery of nucleosome disassembly as demonstrated by complete degradation of DNA by MNase (Fig. 4*B*, lane 10).

### Nucleosome disassembly by AtNuc-L1 requires RNA molecules

**Figure 5:**
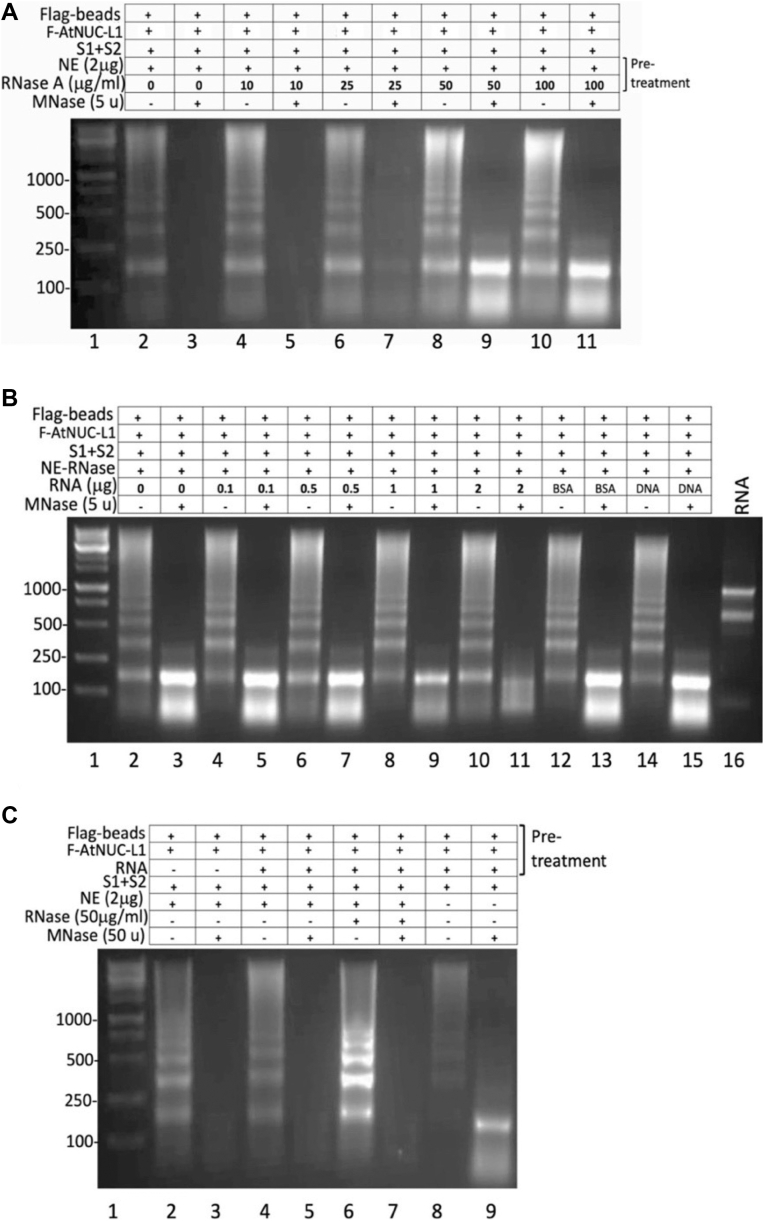
**RNA is required for AtNuc-L1 nucleosome disassembly activity.***A*, pretreatment of NE with RNase A abolished nucleosome disassembly by AtNuc-L1. NE was pretreated with increasing concentrations of RNase A (lanes 4–11). Note, at 50 μg/ml RNase A, nucleosome disassembly was completely abolished. *B*, addition of total tobacco RNA to NE-RNase facilitates the recovery of nucleosome disassembly (lane 11). Bovine serum albumin (2 μg) and salmon sperm DNA (1 μg) were added as controls (Lanes 12–16). *C*, RNase A has no effect on nucleosome disassembly when Flag-AtNuc-L1 pretreated with RNA (lanes 6, 7).

Nucleolins are conserved across animals, plants, and fungi and are characterized by RRMs, an abundant domain in eukaryotes, which is required for binding RNA (27). Plant and yeast nucleolins commonly possess two RRMs while mammalian nucleolins have four RRMs (10). We thus addressed the requirement for RNAs for activation of AtNuc-L1 toward nucleosome disassembly. To this end, NE was pretreated with RNase A and subjected to nucleosome disassembly assay. The results showed (Fig. 5*A*) that nucleosome disassembly was gradually abolished with increasing RNase A concentration. Thus, NE pretreated with 10 or 25 μg/ml RNase A had no or slight effect on nucleosome disassembly (Fig. 5*A*, lanes 5 and 7), while pretreatment of NE with 50 μg/ml of RNase A completely abolished nucleosome disassembly by AtNuc-L1, leading to recovery of DNA (Fig. 5*A*, lane 9) suggesting that RNAs are required for nucleosome disassembly.

We complemented these experiments by adding RNA to the RNase A pretreated NE. Thus, RNase A–treated NE (NE-RNase) was supplemented with various concentrations of total RNAs extracted from tobacco plants (Fig. 5*B*, lane 16) and subjected to nucleosome disassembly assay. We observed nearly full recovery of nucleosome disassembly following addition of 2 μg of tobacco RNAs (Fig. 5*B*, lane 11), further supporting the requirement of RNAs for AtNuc-L1 activity; addition of bovine serum albumin or salmon sperm DNA did not recover nucleosome disassembly (Fig. 5*B*, lanes 13 and 15). Finally, AtNuc-L1 was pretreated with RNA prior to addition of RNase A. The rationale was that interaction of RNA with AtNuc-L1 will protect RNA from digestion by MNase and thus retaining nucleosome disassembly.

Results showed that pretreatment of AtNuc-L1 with RNA maintained nucleosome disassembly in the presence of RNase A (Fig. 5*C*, lane 7), further confirming the requirement for RNA molecules for AtNuc-L1 activity.

## Discussion

The study of histone H2B variants and their role in chromatin structure and function is expanding in recent years. To gain insight into the function of the class I H2B.9 variant, we conducted GFP-TRAP analysis using nuclear extract derived from transgenic plants expressing H2B.9-GFP (9). This analysis uncovered the interaction of histone H2B.9 with various nuclear proteins, which include chromatin remodeling factors, splicing factors, transcription factors, excision repair proteins, histone chaperones, and nucleolar proteins. This emphasizes histone H2B.9 as a central hub that regulates multiple nuclear reactions.

Focusing on the nucleolar histone chaperone AtNuc-L1, we showed that it interacts with histone H2B, but not other core histones, and is capable of interacting with H2B in the context of intact nucleosomes. Moreover, we demonstrated that AtNuc-L1 plays a role in the nucleosome disassembly, and this function requires additional factors found in the nuclear extract. Its activity in nucleosome remodeling is consistent with its involvement in nucleosome sliding (28). Notably, nucleosome sliding and nucleosome disassembly are distinct processes that require ATP-dependent chromatin remodelers. In sliding, the nucleosome is repositioned along the DNA molecule, and the histone octamer is maintained, but in nucleosome disassembly, the octamer is not preserved and disintegrates, often by the removal of H2A/H2B dimers (29). Given that we have used naturally occurring nucleosomes obtained from the S1 and S2 fractions, which are referred to as active and inactive chromatin, respectively (30), it seems that the interaction of AtNuc-L1 to H2B and its role in nucleosome disassembly may not be affected by specific modified H2Bs; nevertheless, this requires further investigation. Nucleosome disassembly is a process whereby the basic structural unit of chromatin breaks down into its components, namely, DNA and core histone proteins. Nucleosome disassembly is crucial for activation of multiple nuclear functions, including DNA replication, gene transcription, and DNA repair (31, 32, 33). It has been presumed that AtNuc-L1 interacts specifically with transcriptionally active rRNA genes, which may be necessary to position nucleosomes in particular transcriptional frames that dictate the “on” or “off” state of transcriptionally active rRNA genes (34). Our data may support this proposition and suggest a mechanistic explanation for the function of AtNuc-L1 in facilitating not only nucleosome sliding (28) but also disassembly of nucleosomes to allow RNA PolI to perform its function in rRNA gene transcription. Another histone chaperone–associated with H2B.9 is the nucleosome assembly protein 1;2 (NAP1;2, At2g19480) that is involved in homologous recombination in *Arabidopsis thaliana* (35). Similarly to AtNuc-L1, NAP1 was shown to be required for nucleosome disassembly *in vitro* (36) and the yeast NAP1 is involved in histone H2A-H2B exchange and assists nucleosome sliding, a process that requires ATP-dependent chromatin remodeling complexes (37).

The activity of AtNuc-L1 in nucleosome disassembly required additional factors found in the nuclear extract. Certain factors depend on ATP for their functionality, since pretreating the nuclear extract with apyrase, which breaks down ATP, eliminated the nucleosome disassembly activity typically triggered by AtNuc-L1. Conceivably, these factors might be related to an ATP-dependent SWI/SNF chromatin/nucleosome remodeling complex that play a major role in controlling chromatin structure and gene transcription in eukaryotic cells (38). The GFP-TRAP analysis of H2B.9-GFP–expressing plants revealed potential chromatin remodeling factors, namely, the SWI3A and SWI3B, components of an ATP-dependent chromatin-remodeling complex required for proper embryo development (39). Other factors include the chromatin remodeling 4 (CHR4, At5g44800) implicated in DNA damage response and homologous recombination (40) and the chromatin remodeling protein Early Bolting In Short Days (EBS) implicated as a negative regulator of flowering time (41). Notably, the Gly-Arg (GAR)–rich C-terminal domain of pea nucleolin was reported to be DNA helicase, which requires ATP and Mg^++^ for its activity (42). Thus, besides its function in nucleosome disassembly, nucleolin could act in unwinding the DNA double helix to assist gene transcription (43, 44).

Interestingly, our data showed that RNA molecules play critical role in nucleosome disassembly mediated by AtNuc-L1, since removal of RNA by RNase A abolished this activity while addition of RNA to RNase A–treated NE restored the activity. This suggests that binding of RNA to AtNuc-L1 RRMs might be necessary for the activation of AtNuc-L1 toward nucleosome disassembly. We presume that AtNuc-L1 may undergo conformational changes upon binding of RNA molecules with its RRMs that allows its function in facilitating nucleosome disassembly. Indeed, structural analysis of RRM/RNA complex showed that the association of two RRMs mediated by RNA molecule could induce conformational changes (reviewed in (45)). The most known protein activated by RNA (dsRNA) is the protein kinase R, an interferon-induced kinase that phosphorylates the eukaryotic initiation factor 2α resulting in the inhibition of protein synthesis (46, 47). Another RNA-activated enzyme is the CRISPR-associated protease CsX29 that undergoes structural rearrangements upon binding to target RNA (48).

The nucleosome disassembly activity of AtNuc-L1 resembles the activity of the mammalian nucleolin 1 (16) and is consistent with the findings that nucleolin 1 plays an important role in the gene transcription of rRNA genes (34, 49). Notably, some reports highlighted the dual role of nucleolin in chromatin transcription, as it is involved in maintaining the euchromatic state of rRNA gene loci, facilitating RNA polymerase I transcription, and also in the repression of rRNA gene transcription (23, 34).

## Experimental procedures

### Construction of plasmids and production of Flag-AtNuc-L1 protein

The Flag-AtNuc-L1 DNA fragment was amplified by PCR using primers Flag-Nuc1-F, 5′ TAAGAAGGAGATATACCATGGGGATGGACTACAAAGACGATGACGACAAGATGGGAAAGTCTAAATCCGCCACC, and Nuc1-R, 5′ ACGGAGCTCGAATTCGGATCCCTACTCGTCACCGAAGGTAGTCTTC, and 100 ng of cDNA using the high-fidelity PCR PrimeSTAR Max DNA Polymerase (TaKaRa Bio). The PCR fragment was run on 1% agarose gel to confirm the fragment size, and the PCR fragment was cleaned using a PCR cleanup kit (Real Genomics). The parental plasmid pET-28a was linearized by digesting with *Nco*I and *Bam*HI restriction enzymes, run on agarose gel, and purified using a gel extraction kit Gel extraction kit (Real Genomics, #YDF100) according to the manufacturer’s protocol. The amplified Flag-AtNuc-L1 fragment was then subcloned into the linearized pET-28a vector by using the infusion cloning kit (TaKaRa) to generate the pET-28a-Flag-AtNuc-L1 construct. The pET28a-Flag-AtNuc-L1 construct was transformed into competent T-10 bacterial cells and plated on LB agar containing ampicillin (100 mg/L). Colony PCR was performed with primers pET28a-F (GCTAGTTATTGCTCAGCGG) and pET28a-R (ATGCGTCCGGCGTAGAGG) to identify positive clones. Plasmid DNA was prepared from positive clones using a Presto mini plasmid kit (Geneaid) and the integrity of the inserted sequence was verified by DNA sequencing. To induce protein expression, a starter culture of a positive clone was grown in LB medium supplemented with ampicillin (100 mg/L) at 37 °C. Upon reaching an OD_600_ of 0.6, IPTG was added to a final concentration of 0.3 mM and the culture was further incubated for 3 h at 37 °C. Cells were harvested by centrifugation (3500 rpm, 15 min) and resuspended in ice-cold NETN buffer (100 mM NaCl, 1 mM EDTA, 20 mM Tris, pH 8.0, and 0.5% NP-40), sonicated, and then centrifuged (10,000 × rpm, 10 min, 4 °C), and the supernatant was collected for further purification steps.

### Arabidopsis growth conditions and nuclear protein extraction

Seeds of transgenic Arabidopsis expressing H2B.9-GFP (9) and of transgenic Arabidopsis plants expressing the nuclear-localized roGFP2-ORP1 (50) obtained from Simon Barak laboratory were sown on standard gardening soil composed of a 2:1 peat-perlite mixture, stratified for 2 days at 4 °C, and transferred to the growth room. Plants were grown in a growth room under controlled growth conditions (photoperiod light:dark 12 h:12 h; temperature 24 ± 2). After reaching the 8 to 9 leaf stage, approximately 6 g of leaves were collected and ground in Nuclei Isolation Buffer containing 10 mM MES.KOH (pH 5.5), 0.2 M sucrose, 2.5 mM EDTA, 2.5 mM DTT, 0.1 mM spermine, 10 mM NaCl, 10 mM KCl, and 0.15% Triton X-100. The homogenate was gently stirred for 1 h at 4 °C and then filtered through 100 μm and 30 μm nylon mesh. The filtrate was centrifuged at 2000 RPM for 8 min at 4 °C. The pellet was washed to remove chloroplasts, and the nuclei were resuspended in low salt, NETN buffer (100 mM NaCl, 1 mM EDTA, 20 mM Tris, pH 8.0, and 0.5% NP-40) supplemented with protease inhibitor cocktail (Sigma). Nuclei were sonicated on ice three times, 1 min each (1 min interval) at 65% amplitude to disrupt the nuclear envelope. The sonicated samples were centrifuged, and the supernatant containing the soluble nuclear proteins (NE) was collected and stored at −20 °C for further analysis.

### GFP Trap and proteome analysis

GFP-TRAP was performed on total nuclear proteins extracted (NE) from Arabidopsis transgenic plants expressing H2B.9-GFP; as a reference, we used nuclear extract from nuclear-localized roGFP2-ORP1 (3 replicates each) using GFP-Trap agarose (Chromotek) according to the manufacturer’s protocol. Precipitated proteins were subjected to proteomic analysis.

Proteome analysis was performed by the proteomic services of The Smoler Protein Research Center at the Technion. Proteins were digested with trypsin, followed by separation and mass measurement *via* liquid chromatography with tandem mass spectrometry on LTQ Orbitrap (ThermoFisher Scientific; https://proteomics.net.technion.ac.il/proteomic-services/ accessed on 11 May 2021). Protein identification and quantification were done using MaxQuant, using *A. thaliana* proteins from UniProt as a reference. All the identified peptides were filtered with high confidence, top rank, and mass accuracy. High-confidence peptides passed the 1% FDR threshold (FDR = false discovery rate, the estimated fraction of false positives in a list of peptides). A protein was considered “present” if it occurred in at least two replicates of the H2B.9-GFP as compared to the reference roGFP2-ORP1 alone with zero values in all replicates and it is represented by at least two peptides. Nuclear proteins were selected based on the UniProt annotation for cellular location.

### FLAG pull-down, histone preparation, immunoblotting, and co-immunoprecipitation

Expression in bacteria and purification of Flag-fusion protein was performed by Flag pull-down essentially as described (51). Thus, Flag-AtNUC-L1 immobilized onto anti-FLAG M2 magnetic beads (Merck) was incubated either with total nuclear proteins (NE) extracted from H2B.9-GFP Arabidopsis leaves or with nucleosomes (chromatin fractions, S1 and S2) obtained from tobacco by chromatin fractionation (52), calf thymus histones preparation (Worthington), or from *Nicotiana tabacum* leaves. Following extensive washing with NETN, precipitated proteins were subjected to 15% SDS/PAGE and immunoblotted.

Histones (acid soluble fraction) were prepared from tobacco leaves in 3% trichloroacetic acid in NETN buffer supplemented with protease inhibitor cocktail (Sigma) essentially as described (53). Protein concentration was determined by the Bradford reagent. Acid soluble proteins (2.5 μg) enriched with histones were resolved by 15% SDS/PAGE gel, stained with Ponceau, and immunoblotted.

Immunoblotting of precipitated proteins was performed as follows. Briefly, proteins were separated on 15% SDS/PAGE, transferred onto nitrocellulose membrane, stained with Ponceau, and probed with antibodies to histone proteins, anti-Flag, or anti-GFP. Antibodies to histone H3 (D1H2), H4 (L64C1), and H2B (D2H6) were purchased from Cell Signaling, while the H2A (PHY0861A) antibody was purchased from PhytoAB. Antibodies to FLAG were from Agrisera (AS152871) and anti-GFP (A01388-40) from Genscript. Immunodetection was performed using a secondary antibody of goat anti-rabbit alkaline phosphatase or goat anti-mouse alkaline phosphatase conjugate (Sigma) and BCIP/NBT substrate (Roche).

Co-immunoprecipitation reaction mixture contains 2 μg of anti H2B immobilized on 25 μl of protein A-agarose (GeneScript, recombinant protein A of about 34 kDa), 2 μg of calf thymus histone proteins, and 500 μl of bacterial extract containing Flag-AtNuc-L1. Samples were incubated at 4 °C for 4 h followed by three washes with NETN and bound proteins were run on 12% SDS/PAGE and immunoblotted using anti Flag and visualized as described above.

### Nucleosome disassembly assay

We developed an *in vitro* assay to determine the capacity of AtNuc-L1 to disassemble nucleosomes. Accordingly, bacterial lysates (500 μl) containing Flag-AtNuc-L1 was first immunoprecipitated using 15 μl of anti-Flag M2 magnetic beads (Merck). The Flag-AtNuc-L1–bound beads were then incubated at room temperature for 2 h with 300 μl of nucleosomes (chromatin fractions, S1 and S2) obtained from tobacco by chromatin fractionation (52). Then 2 μg of NE from *N. tabacum* was added and incubation continued for another 20 min. Following incubation, half of the reaction mixture (150 μl) was treated with 5 to 50 units of micrococcal nuclease (MNase, New England Biolabs, #M0247S) for 5 min at 37 °C; the reaction was stopped by adding 350 μl of stop solution (2 mg/ml proteinase K, 10 mM NaCl, 1 mM MgCl_2_, 10 mM Tris–HCl pH 7.5, 2% SDS). The remaining half of the reaction mixture, which was used as a control, was treated with 350 μl of stop solution and all samples were incubated overnight at 37 °C. Following incubation, each sample was added 140 μl of 5 M potassium acetate, mixed gently by inversion, and incubated on ice for 15 min followed by centrifugation at 14,000 rpm for 15 min at 4 °C. The supernatant was collected and extracted once with chloroform:isoamyl alcohol (24:1), and the upper aqueous phase was recovered. DNA was precipitated by adding 1 ml of ice-cold 100% ethanol, kept in −20 °C overnight, and centrifuged to pellet the DNA. The pellet was washed, air-dried, and resuspended in 20 μl of TE buffer (10 mM Tris–HCl pH 8.0, 1 mM EDTA). DNA samples were resolved on a 1.6% agarose gel and visualized by staining with ethidium bromide. If nucleosome disassembly occurs, DNA will be separated from core histone proteins and be subjected to degradation by MNase, resulting in no recovery of DNA. As controls for the nucleosome disassembly assays, we used extract (500 μl) derived from bacteria containing the parental pET28a as well as Flag-KLF4 protein provided by Giritharan Jagadeesan (Dani Levy’s lab).

### Apyrase-treated NE assays

To evaluate whether nucleosome disassembly by AtNuc-L1 requires ATP, Flag-TRAP–immobilized AtNuc-L1 was incubated with S1 + S2 nucleosomes and nuclear extract pre-treated with 0.5, 1, or 2 units of apyrase ((#M0398S, New England Biolabs) for 30 min at room temperature to deplete endogenous ATP. The reaction mixture was incubated for 20 min at room temperature, followed by the addition of 5 units of MNase and further incubation for 5 min at 37 °C.

To test whether exogenous ATP could restore nucleosome disassembly activity, different concentrations of ATP (Sigma, 0.25, 0.5, 1, and 2 mM) were added to reactions containing NE pretreated with apyrase. After incubation, reactions were stopped by adding 350 μl of stop solution, and DNA was extracted and analyzed on 1.6% agarose gels stained with ethidium bromide.

### RNase A–treated NE assays

To assess the involvement of RNA in nucleosome disassembly, NEs were pretreated with RNase A (10, 25, 50, and 100 μg mL^−1^; Thermo Fisher scientific, #EN0531) prior to incubation with Flag-TRAP–immobilized AtNuc-L1 and S1 + S2 nucleosomes. To determine whether exogenous RNA could restore disassembly activity, different concentrations of total RNA (0.1, 0.5, 1, and 2 μg) extracted from tobacco leaves (RNA extraction kit, Yeasen Biotech) were added to the NE that had been pretreated with RNase A (50 μg mL^−1^). The RNA-supplemented NE was then added to the reaction mixture containing AtNuc-L1 and nucleosomes. As negative controls, 1 μg bovine serum albumin and 1 μg salmon sperm DNA were added instead of RNA. After incubation at room temperature for 20 min, 5 U MNase was added, and the reaction was allowed to proceed for 5 min. The reactions were stopped by adding stop solution; DNA was extracted and analyzed on 1.6% agarose gels stained with ethidium bromide.

## Data availability

All data are included in the article and/or its Supporting information files.

## Supporting information

This article contains supporting information.

## Conflicts of interest

The authors declare that they have no conflicts of interest with the contents of this article.
