# Supplementary material for: Histone H2B-associated proteins: The Arabidopsis nucleolin 1 binds H2B and facilitates nucleosome disassembly via RNA-dependent mechanism
Source: J Biol Chem. 2026 May 11;302(6):113137. doi: 10.1016/j.jbc.2026.113137 (PMC13266012; doi:10.1016/j.jbc.2026.113137)
Supplement: Supporting Figures [file mmc1.docx]

**Supporting information / Figures**

Histone H2B-associated proteins: the Arabidopsis nucleolin 1 binds H2B and facilitates nucleosome disassembly via RNA-dependent mechanism

Yarra et al.


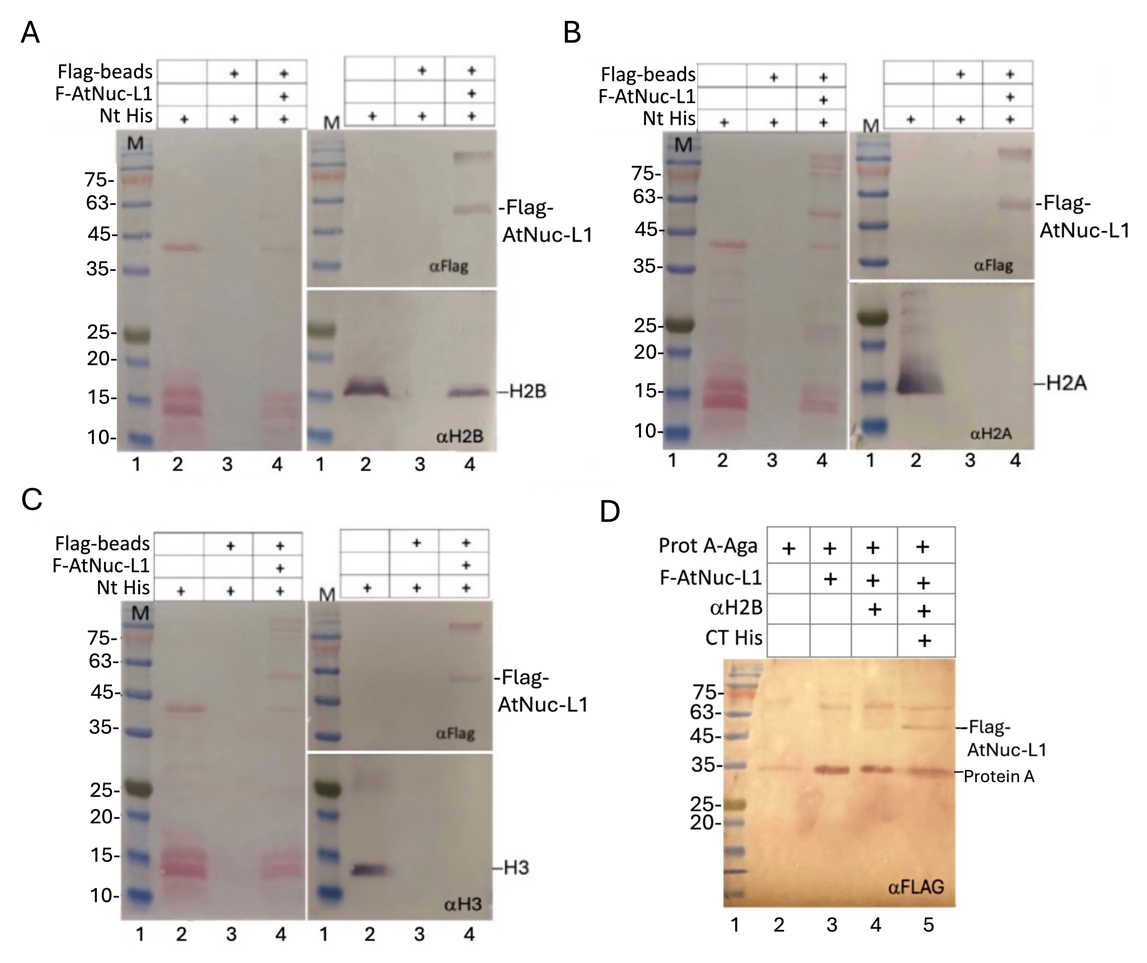


Fig. S1. The AtNuc-L1 protein specifically interacts with H2B from tobacco histone preparation. (A-C) Flag-AtNuc-L1 immobilized on Flag beads was incubated with histone proteins prepared from tobacco leaves (Nt His). Bound proteins were separated on 12% SDS/PAGE, transferred onto membrane and stain with Ponceau (left panels in A-C), and immunoblotted (right panels in A-C) with anti H2B (A), anti H2A (B) and anti H3 (C). Lane 2 in panels A to C indicates the input histones. (D) Reciprocal experiment demonstrating that anti H2B immobilized on protein A-agarose (Prot A-Aga) in the presence of histones precipitated Flag-AtNuc-L1 (Lane 5). Lane 2 is the input protein A-agarose. Protein A of about 34 kDa that leached from the beads is indicated.


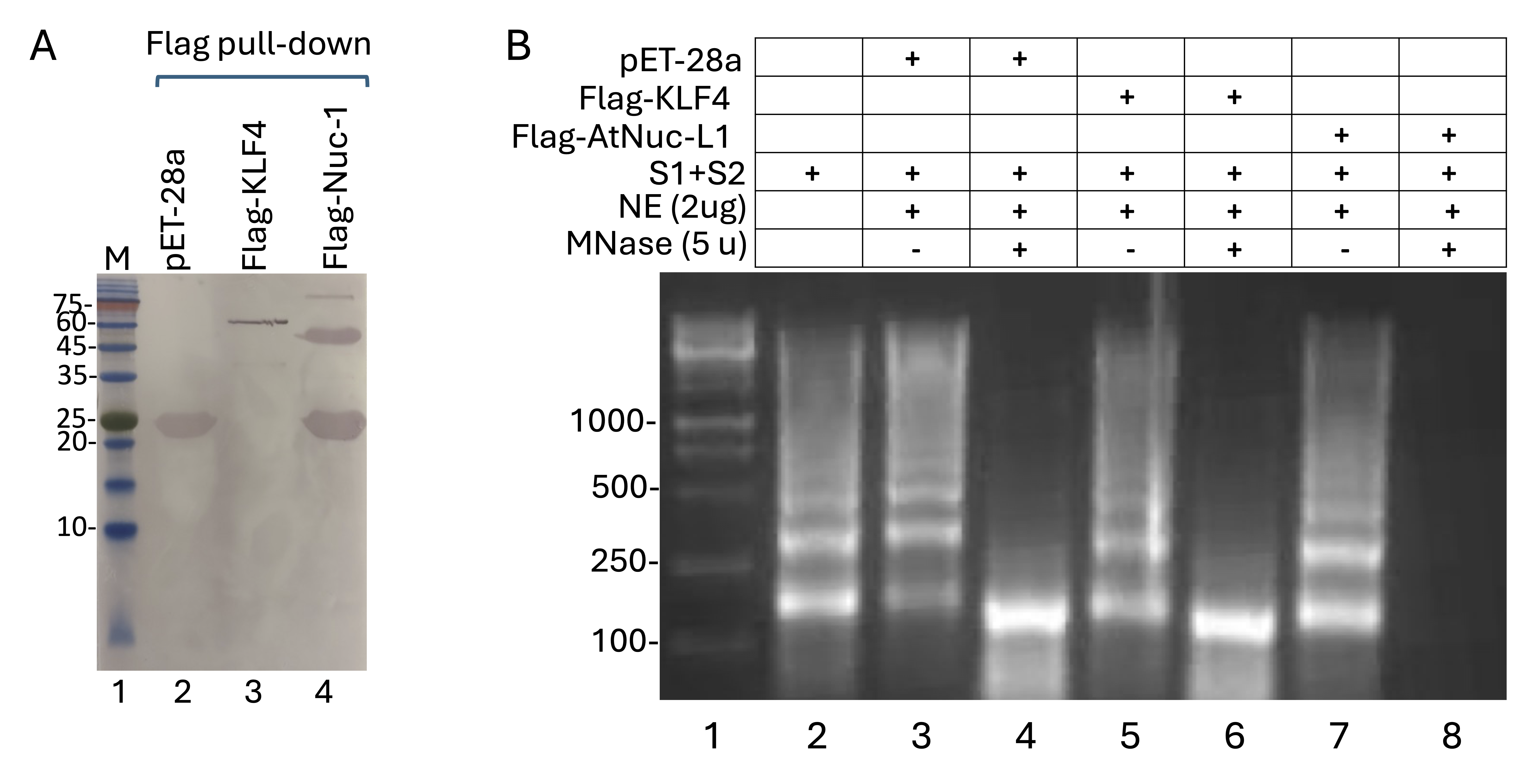


Fig. S2. Nucleosome disassembly is facilitated by AtNuc-L1. (A) Flag pull-down from pET28a, Flag-KLF4 and Flag-AtNuc-L1 bacterial extracts. Note, the 25 kDa protein co-purified on Flag beads is of bacterial origin. (B) AtNuc-L1 facilitates nucleosome disassembly. Reaction mixture containing co-purifying proteins (pET28a, lanes 3 and 4), Flag-KLF4 (lanes 5 and 6) or Flag-AtNuc-L1 (Lanes 7 and 8) were subjected to nucleosome disassembly assays with the indicated components. Note, no activity is evident with pET28a copurifying proteins or with Flag-KLF4. Lane 2 is the input S1+S2 fractions.


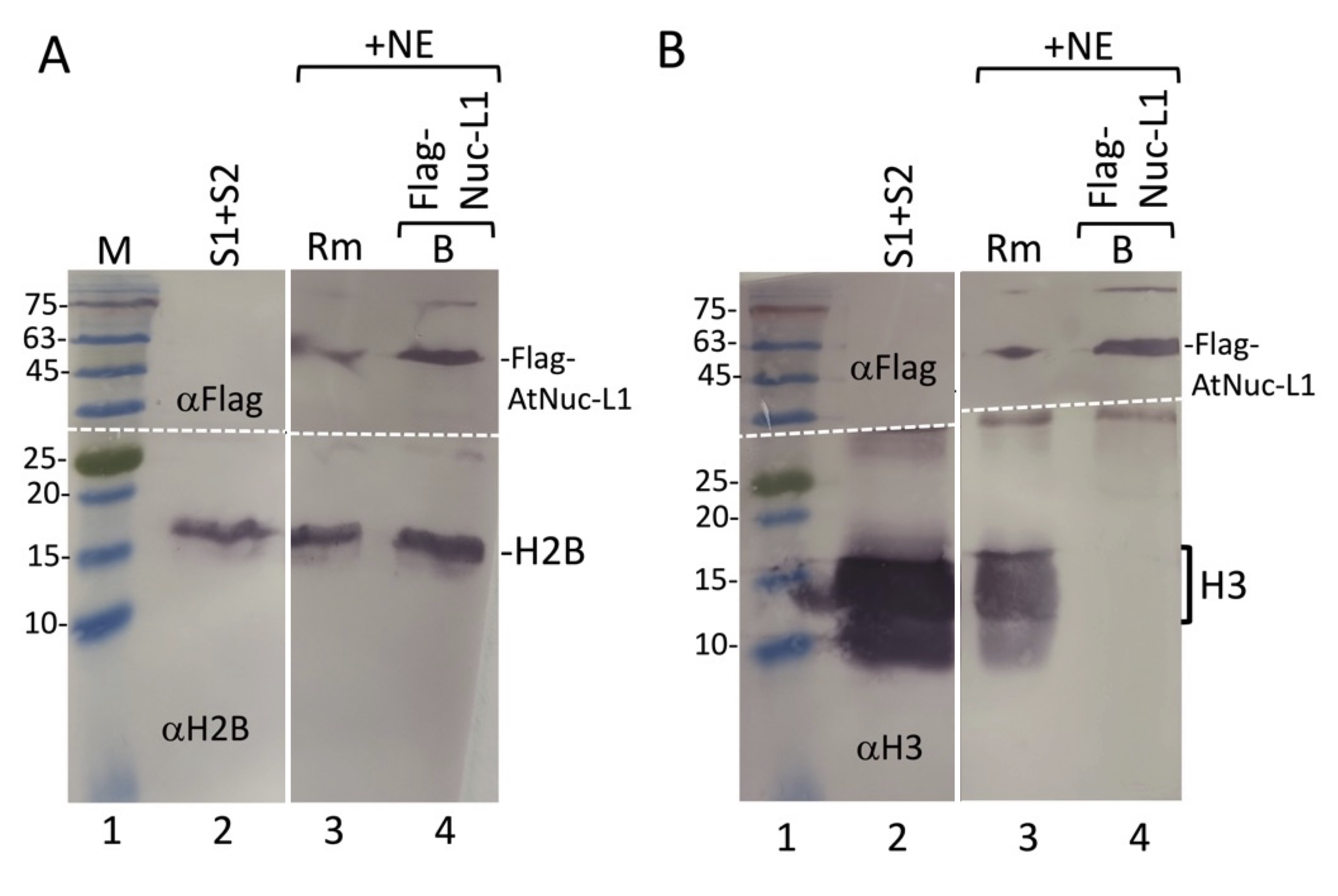
Fig. S3. Confirmation of nucleosome disassembly by AtNuc-L1. Reactions were conducted in the presence of S1+S2 and Flag-AtNuc-L1 with nuclear extract (+NE). The reaction samples (lanes 4 in A and B) were loaded onto Flag-AtNuc-L1 immobolized on Flag beads and bound (B) proteins were separated on 12% SDS/PAGE, tranferred onto membrane and immunoblotted. Lanes 2 in A and B are the input S1+S2 fractions, and lanes 3 are the input reaction mixture (Rm). Note, each membrane in A and B was cut above the 25 kDa (broken line) to probe the upper part and the lower part with anti-Flag and anti-H2B (A) or anti H3 (B), respectively.
